# Dimorphic-root ideotype demonstrates increased rice yield in nutrient-deficient paddy fields in Madagascar

**DOI:** 10.1093/jxb/erag043

**Published:** 2026-01-27

**Authors:** Toshiyuki Takai, Tsiry Rakotoarinjara, Tefy Manantsu Rajaonera, Viviane Raharinivo, Aung Zaw Oo, Tomohiro Nishigaki, Hidetoshi Asai, Yojiro Taniguchi, Yuka Kitomi, Shota Teramoto, Yasuhiro Tsujimoto, Yusaku Uga

**Affiliations:** Crop, Livestock and Environment Division, Japan International Research Center for Agricultural Sciences (JIRCAS), Tsukuba, Ibaraki 305-8686, Japan; Rice Research Department, National Center of Applied Research on Rural Development, Tsimbazaza, Antananarivo BP1690, Madagascar; Rice Research Department, National Center of Applied Research on Rural Development, Tsimbazaza, Antananarivo BP1690, Madagascar; Rice Research Department, National Center of Applied Research on Rural Development, Tsimbazaza, Antananarivo BP1690, Madagascar; Crop, Livestock and Environment Division, Japan International Research Center for Agricultural Sciences (JIRCAS), Tsukuba, Ibaraki 305-8686, Japan; Crop, Livestock and Environment Division, Japan International Research Center for Agricultural Sciences (JIRCAS), Tsukuba, Ibaraki 305-8686, Japan; Crop, Livestock and Environment Division, Japan International Research Center for Agricultural Sciences (JIRCAS), Tsukuba, Ibaraki 305-8686, Japan; Institute of Crop Science, National Agriculture and Food Research Organization (NARO), Tsukuba, Ibaraki 305-8518, Japan; Institute of Crop Science, National Agriculture and Food Research Organization (NARO), Tsukuba, Ibaraki 305-8518, Japan; Institute of Crop Science, National Agriculture and Food Research Organization (NARO), Tsukuba, Ibaraki 305-8518, Japan; Crop, Livestock and Environment Division, Japan International Research Center for Agricultural Sciences (JIRCAS), Tsukuba, Ibaraki 305-8686, Japan; Institute of Crop Science, National Agriculture and Food Research Organization (NARO), Tsukuba, Ibaraki 305-8518, Japan; National Institute of Agricultural Botany, UK

**Keywords:** *DRO1*, nutrient-deficient paddy fields, *qsor1*, rice, root ideotype, root system architecture (RSA), yield

## Abstract

Demand for rice (*Oryza sativa* L.) is rapidly increasing in sub-Saharan Africa; however, its productivity is constrained by nutrient-poor paddy soils, particularly those deficient in N and P. Improving root system architecture offers potential to enhance nutrient uptake efficiency and increase yield under such conditions. This study aimed to identify the most effective root phenotype for enhancing grain yield under the nutrient-deficient soil conditions in Madagascar. A high-yielding *indica* cultivar—IR64—and 12 near-isogenic and pyramiding lines carrying single or multiple quantitative trait loci for root growth angle, length, volume, and thickness were evaluated across several nutrient-deficient paddy fields with varying nutrient gradients with depth. A pyramiding line combining *DRO1* and *qsor1*, which confer deeper and shallower rooting traits, respectively, exhibited significantly higher grain yield than IR64 at the yield levels of 2.7–5.1 t ha^−1^. This dimorphic root system expanded root distribution into both topsoil and subsoil, enhanced N and P uptake, and increased overall grain yield by 16–23% relative to IR64. These findings suggest that root system architecture expansion into multiple soil layers represents a promising ideotype for improving rice performance in nutrient-deficient paddy fields and may serve as a key breeding target to boost rice production in sub-Saharan Africa.

## Teaser

In rice, combining the deep-root allele *DRO1* and shallow-root allele *qSOR1* expands root distribution into both topsoil and subsoil layers, enhancing yield in nutrient-poor paddy fields in Madagascar.

## Introduction

Rice (*Oryza sativa* L.) has emerged as a key staple food in sub-Saharan Africa (SSA), fueled by population growth and shifting consumer preferences (Balasubramanian *et al*., 2007). However, despite rising demand, the average rice yield in SSA remains low at 2.3 t ha^−1^, less than half the average yield of 5.0 t ha^−1^ in Asia (FAOSTAT, 2025). One of the primary constraints to rice productivity in SSA is low soil fertility (Tittonell and Giller, 2013). Approximately 60% of the rice-growing area in the region is characterized by highly weathered soils with very limited indigenous nutrient supply and poor nutrient retention capacity (Haefele *et al*., 2014). Among the essential nutrients N, P, and K, N is the most limiting, followed by P (Saito *et al*., 2019). The use of chemical fertilizers offers a viable strategy to improve yields in these nutrient-deficient soils. However, recent increases in global fertilizer prices due to unstable global situations have rendered them less accessible to smallholder farmers in SSA (World Bank, 2025). Consequently, fertilizer use efficiency and developing rice cultivars adapted to nutrient-deficient conditions are critical for enhancing rice productivity in the region (Ismail *et al*., 2007; Tsujimoto *et al*., 2019; Rakotoson *et al*., 2022).

Rice plants deficient in N and/or P exhibit stunted growth and significantly reduced tillering (Dobermann and Fairhurst, 2000). The suppression of axillary bud outgrowth under P deficiency is considered an adaptive strategy to optimize shoot architecture and conserve limited P resources (Umehara *et al*., 2010). However, excessive inhibition of tillering may limit yield potential in nutrient-deficient soils (Takai, 2024; Takai *et al*., 2024b). Tsujimoto *et al*. (2020) reported that a rice cultivar with vigorous tillering traits maintained high biomass production under P-deficient conditions. More recently, *MP3*, a natural allele of *OsTB1/FC1*—previously identified as a negative regulator of tiller bud outgrowth—was found to moderately enhance tillering and increased the panicle number in nutrient-deficient paddy fields in Madagascar (Takai *et al*., 2021, 2023). Additionally, genome editing of *OsTB1/FC1* slightly increased tiller/panicle number, leading to improved grain yield under P deficiency (Ishizaki *et al*., 2023). These findings suggest that improved aboveground plant architecture contributes to increased rice productivity in nutrient-deficient soils.

In addition to aboveground traits, underground plant architecture plays a critical role in nutrient and water acquisition, with roots being essential for absorbing these resources from the soil (Den Herder *et al*., 2010; Uga, 2021). In upland fields, N is highly mobile and tends to leach in the subsoil along with water. Considering these characteristics, the ‘steep, cheap, and deep’ root ideotype was proposed for maize to enhance N and water uptake from the subsoil (Lynch, 2013). Notably, this ideotype proved effective for rice in nutrient-rich paddy fields, where a deep-rooting rice line exhibited higher N uptake and grain yield than a shallow-rooting line (Arai-Sanoh *et al*., 2014). However, whether the ideotype is also effective in N-deficient paddy fields remains unclear. In contrast, P is relatively immobile and accumulated primarily in the topsoil of upland fields. Accordingly, root traits such as shallower axial root angles, increased adventitious rooting, a higher number of axial roots, and a broader lateral root dispersion have been suggested as optimal ideotypes for enhancing P acquisition in P-deficient soils, particularly in crops like bean and maize (Lynch, 2011). In contrast, in rice, Rose *et al*. (2013) reported that the topsoil of paddy fields might not be enriched in P since paddy fields lack a structured soil profile owing to repeated puddling. A recent study in the central highlands of Madagascar also demonstrated no abundant P accumulation in the top layer, as the paddy fields had received minimal fertilizer input (Oo *et al*., 2025). Considering that both N and P are often deficient in the paddy fields in SSA and that the nutrient gradients differ from those in upland fields, identifying root phenotypes that optimize the acquisition of both nutrients is essential (Oldroyd and Leyser, 2020; Rangarajan *et al*., 2022). However, to date, no study has systematically investigated such optimal root phenotypes in rice cultivated under the nutrient-deficient paddy field conditions.

The completion of whole-genome sequencing of rice (International Rice Genome Sequencing Project, 2005), along with advances in genotyping and phenotyping technologies, has facilitated the identification of quantitative trait loci (QTLs) associated with root system architecture (RSA) (Courtois *et al*., 2009; Uga *et al*., 2015). Among the large number of RSA-related QTLs (Courtois *et al*., 2009; Daryani *et al*., 2022), several have been fine-mapped and cloned as single genes, including those controlling root growth angle (*DRO1* and *qSOR1*; Uga *et al*., 2013a; Kitomi *et al*., 2020), root length (*qRL6.1*; Obara *et al*., 2010), root volume (*qFSR4*; Ding *et al*., 2011), and root thickness (*Sta1*; Uga *et al*., 2010). The application of such QTLs offers a promising approach to define optimal RSA traits for improving rice productivity in nutrient-deficient paddy fields, particularly in SSA.

In this study, we aimed to identify the most effective root phenotype for enhancing grain yield under the nutrient-deficient soil conditions of the central highlands of Madagascar (Nishigaki *et al*., 2019; Tsujimoto *et al*., 2019). As observed in SSA, rice productivity in Madagascar is also constrained by the predominance of highly weathered soils such as Ferralsols (Rakotoson *et al*., 2022), and the average rice yield is 2.5 t ha^−1^, comparable to the 2.3 t ha^−1^ average in SSA countries (FAOSTAT, 2025). To achieve the above objective, we conducted yield trials using both near-isogenic lines (NILs), harboring individual QTLs, and pyramiding lines (PYLs), carrying multiple QTLs. The findings provide genetic evidence supporting a rice root ideotype for future breeding programs targeting SSA and similarly challenged agroecosystems.

## Materials and methods

### Plant materials

In this study, we selected five QTLs (*DRO1*, *qsor1*, *qFSR4*, *Sta1*, and *qRL6.1*) related to RSA. The functional alleles of *DRO1* and *qSOR1* promote deeper rooting, whereas their non-functional allele (*dro1* and *qsor1*) result in shallower roots (Uga *et al*., 2013a; Kitomi *et al*., 2020). *qFSR4* increases root volume (Ding *et al*., 2011), *Sta1* enhances root thickness (Uga *et al*., 2010), and *qRL6.1* promotes root elongation (Obara *et al*., 2010). Six NILs and six PYLs carrying these QTLs were developed in the IR64 genetic background (Fig. 1). IR64 is a high-yielding lowland *indica* cultivar developed by the International Rice Research Institute, Philippines, in 1985 (Mackill and Khush, 2018). The NILs *DRO1*-NIL, *qFSR4*-NIL, *Sta1*-NIL, and *qRL6.1*[KP]-NIL carry QTLs derived from Kinandang Patong (KP), an upland tropical *japonica* cultivar (Uga *et al*., 2013a; Deshmukh *et al*., 2017; Iba *et al*., 2025). *qsor1*-NIL carries a QTL derived from Gemdjah Beton—an Indonesian lowland rice belonging to the Bulu ecotype (Kitomi *et al*., 2020). *qRL6.1*[Kasalath]-NIL was developed by crossing IR64 with Kasalath—an *indica* cultivar from India, used as the *qRL6.1* donor—followed by backcrossing with IR64 and DNA marker-assisted selection. The following PYLs were developed via crosses between respective NILs: *DRO1*+*qsor1*-PYL, *qFSR4*+*DRO1*-PYL, *DRO1*+*Sta1*-PYL, and *qRL6.1*[KP]+*DRO1*-PYL (Deshmukh *et al*., 2017; Kitomi *et al*., 2020; Iba *et al*., 2025). *qFSR4*+*Sta1*+*DRO1*-PYL and *qRL6.1*[KP]+*Sta1*+*DRO1*-PYL were developed through further crosses between NILs and double QTL-PYLs (Iba *et al*., 2025).

**Fig. 1.**
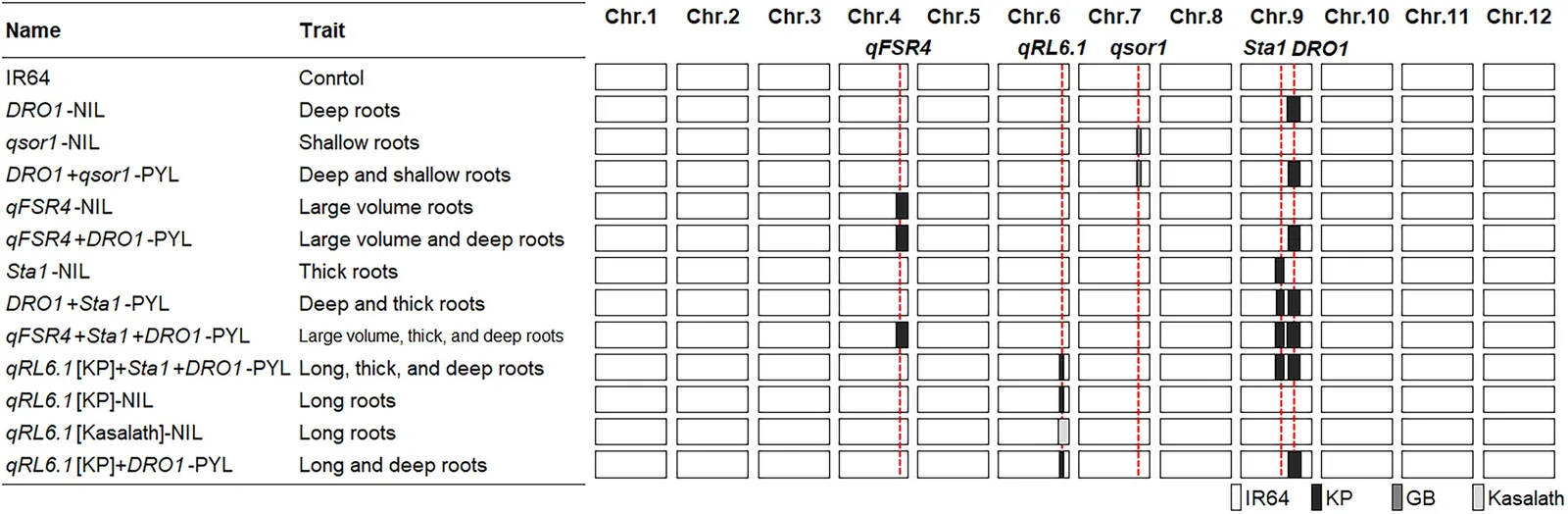
Graphical representation of the genotypes of IR64, near-isogenic lines (NILs), and pyramiding lines (PYLs) used in this study. White rectangles represent chromosome regions derived from IR64. Black, dark gray, and light gray rectangles indicate chromosome segments from Kinandang Patong (KP), Gemdjah Beton (GB), and Kasalath, respectively. Red dashed lines denote the positions of each QTL. *DRO1* promotes deeper rooting (Uga *et al*., 2013a), whereas *qsor1* results in shallower roots (Kitomi *et al*., 2020). *qFSR4* increases root volume (Ding *et al*., 2011), *Sta1* enhances root thickness (Uga *et al*., 2010), and *qRL6.1* promotes root elongation (Obara *et al*., 2010).

### Field experiments and cultivation methods

Field experiments were conducted in farmers’ paddy fields across the central highlands of Madagascar during the 2022–2023 (hereafter, 2023) and 2023–2024 (hereafter, 2024) growing seasons. In 2023, four paddy fields were selected as experimental sites: Site 1 (19°48′ S, 46°45′ E; 1235 m altitude), Site 2 (19°49′ S, 46°48′ E; 1287 m altitude), Site 3 (19°40′ S, 46°34′ E; 1107 m altitude), and Site 4 (19°39′ S, 46°33′ E; 1125 m altitude) (Fig. 2). These sites are located in close proximity and experience similar weather conditions (Supplementary Table S1). At all sites, rice had been cultivated once annually without crop rotation or mineral fertilizer application for at least 10 years prior to the experiment. Soil chemical properties were analysed in 10 cm intervals from a depth of 0 to 50 cm prior to the start of the trials (Fig. 3). Across all sites, the mean organic C and total N content ranged from 8.8 to 17.5 g C kg^−1^ and 0.9 to 1.6 g N kg^−1^, respectively—characteristic of low-organic-matter and N-deficient paddy soils in the central highlands of Madagascar (Asai *et al*., 2021; Rakotonindrina *et al*., 2023). Mean available P (Olsen P) levels ranged from 1.1 to 8.7 mg P kg^−1^, indicating a range from very low to moderate P availability, based on established classification criteria (Dobermann and Fairhurst, 2000). Notably, the total N and available P contents in the subsoil were neither consistently higher nor lower than those in the topsoil. This supports the influence of repeated puddling and little fertilizer application (Rose *et al*., 2013; Oo *et al*., 2025). Seeds of IR64, six NILs, and six PYLs were sown in nursery beds established near each experimental site. Then, 20- to 30-day-old seedlings were transplanted into the fields at a spacing of 20×20 cm, with two seedlings per hill, resulting in a planting density of 25 hills m^−2^. Each plot measured 4–4.8 m^2^ and was arranged in a split-plot design, with the main plot used for fertilizer treatments (with and without fertilizer) and subplots for rice genotypes, replicated four times. Basal fertilizer was applied to the surface designated plots immediately after transplanting, at rates of 20 kg N ha^−1^, 17.5 kg P ha^−1^, and 24.1 kg K ha^−1^, using a N–P–K fertilizer (11–22–16). Plots were top-dressed with urea (20 kg N ha^−1^) at 30 and 60 d after transplanting.

**Fig. 2.**
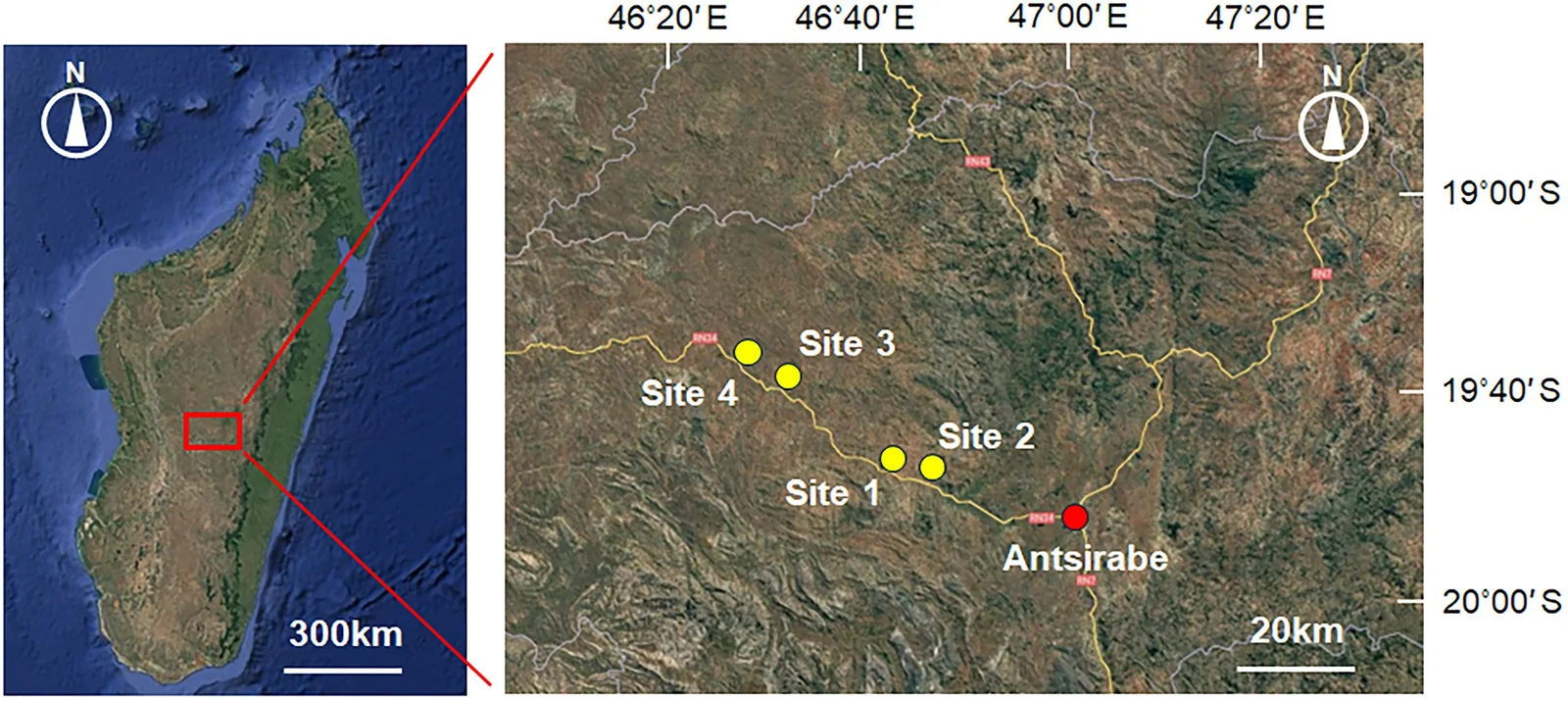
Maps of the four experimental sites located in the central highlands of Madagascar. Map data © Google Earth Pro 7.3.6, OpenGL, Data SIG, Landsat/Copernicus, build date January 2025, server: kh.google.com.

**Fig. 3.**
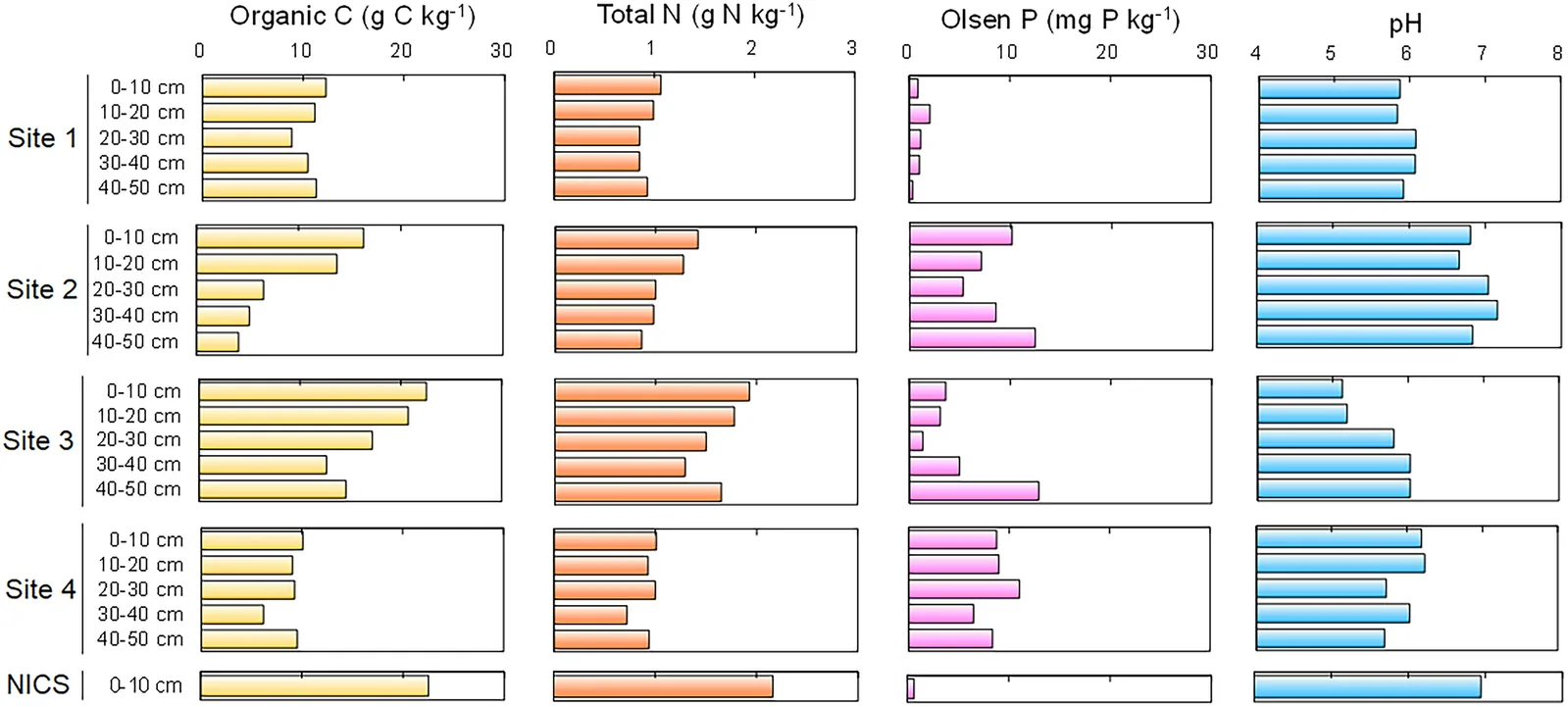
Soil chemical properties measured at 10 cm intervals from 0 to 50 cm depth at each experimental site in Madagascar, and at the surface layer of a paddy field at the National Institute of Crop Science (NICS), Japan, used for the pot trial.

In 2024, field experiments were conducted at Sites 2, 3, and 4, which were the same sites as those used in 2023. The same cultivation methods and experimental designs were applied, with the exception that the plot size in 2024 was 9.6 m^2^. Based on the 2023 results, four rice genotypes—IR64, *DRO1*-NIL, *qsor1*-NIL, and *DRO1*+*qsor1*-PYL—were selected for a more detailed assessment of root phenotypes and nutrient uptake.

### Evaluation of yield and yield components at maturity

In both 2023 and 2024, rice yield and yield components were evaluated at maturity. Panicle number was counted within 1.1–1.6 m^2^ (28–40 hills) per plot. The hills were then harvested for further analyses. After threshing, total grain weight was recorded, and the moisture content was measured using a grain moisture tester (Riceter f512; Kett, Tokyo, Japan). Approximately three sets of 40 g grains were selected as subsamples and counted using an electronic seed counter (WAVER IC-VA; Aidex Co. Ltd., Nagoya, Japan). Spikelet number per square meter was calculated by multiplying number of grains per unit weight in the subsample by the total grain weight per square meter. Spikelet number per panicle was calculated by dividing spikelets per square meter by panicles per square meter. Grain filling was assessed by submerging subsamples in tap water; grains that sank were considered filled. These filled grains were oven-dried at 37 °C to a constant weight, counted, and weighed. Moisture content of the filled grains was also measured. The percentage of filled spikelets was calculated as the ratio of filled spikelets to total spikelets in the subsample. Single-grain weight was calculated by dividing the total weight of filled spikelets by their number. Grain yield was calculated by multiplying the yield components—panicle number per square meter, spikelets per panicle, filled grain percentage, and single-grain weight. Both final yield and single-grain weight were adjusted to 14% moisture content.

### Evaluation of aboveground biomass, N, and P content

In 2024, the number of panicles on 15 hills per plot was counted at maturity. One hill with an intermediate panicle number was selected from the 15 hills. Aboveground shoots were then separately sampled from the selected hill and remaining 14 hills. The sample from the selected hill was further separated into grain and straw fractions. These samples were oven-dried at 70 °C for 3 d and weighed to determine the aboveground biomass. After weighing, grain and straw samples were ground into a fine powder using a mill (IFM-800DGM; Iwatani, Osaka, Japan). N concentrations in grains and straw were determined using an NC analyser (Sumigraph NC-220 F; Sumika Chemical Analysis Service Ltd, Tokyo, Japan). Grain N uptake, straw N uptake, and total aboveground N uptake at maturity were calculated as the products of dry weight and corresponding N concentrations, with total uptake being the sum of grain and straw N uptake. P concentrations in grain and straw were measured using the molybdate blue method following dry-ashing at 550 °C for 2 h and extraction with 0.5 M HCl (Murphy and Riley, 1962). Aboveground P uptake was calculated in the same manner as that for N uptake.

### Evaluation of root growth angle and root biomass

In 2024, root growth angle was evaluated using a modified version of the basket method described by Uga *et al*. (2011). At transplanting, a plastic hemispherical mesh basket (Yazaki Kako, Shizuoka, Japan), with a top diameter of 15 cm, a bottom diameter of 8.5 cm, and a height of 6 cm, was filled with paddy soil and placed in each experimental plot, ensuring that the top surface of the basket was level with the surrounding field soil. Two seedlings were transplanted into each basket. At 60 d after transplanting, the baskets were excavated, and the number of roots penetrating the bottom and sides of the mesh, as well as those emerging from the top surface, were counted. Then, the proportion of roots at each position relative to the total root number was calculated. Roots penetrating the bottom mesh (angle of 53–90° from the horizontal) were defined as deep roots, whereas those emerging from the top surface were classified as shallow roots.

Root biomass was also assessed at 60 d after transplanting using a stainless-steel monolith sampler (15 cm diameter, 30 cm depth) (Daiki Rika Kogyo, Saitama, Japan) placed over a single rice hill with an intermediate tiller number. The sampler was driven into the soil using a rubber hammer, and the resulting soil block was extracted and washed with river water to remove soil. The cleaned root samples were then oven-dried at 70 °C for 3 d and weighed to determine root biomass.

### Three-dimensional imaging of root distribution using X-ray computed tomography

To further assess root distribution in IR64, *DRO1*-NIL, *qsor1*-NIL, and *DRO1*+*qsor1*-PYL, rice plants were grown in pots placed in a greenhouse at the National Institute of Crop Science (NICS), National Agriculture and Food Research Organization, Tsukuba, Japan, in 2024. Glay lowland soils with low P availability (Fig. 3)—similar to those found in Madagascar—were collected from the surface layer of the NICS paddy fields that had not received P fertilizer for over 20 years. The soils were air-dried and sieved using an 8.0 mm mesh. Plastic Wagner pots (1/5000a) (Fujiwara Scientific Co., Ltd, Tokyo, Japan) were filled with 2.7 kg of the prepared soil. A randomized complete block design was used, with four replications across two fertilizer treatments (with and without NPK application) and four genotypes. While thoroughly mixing soils, ammonium nitrate (NH_4_NO_3_), triple super phosphate, and potassium sulfate (K_2_SO_4_) were applied to the NPK-applied pots (half of all pots) at rates of 120 mg N pot^−1^, 350 mg P pot^−1^, and 48 mg K pot^−1^, respectively. Fifteen-day-old seedlings were transplanted into the pots, with one seedling per pot. Greenhouse conditions were maintained at 30 °C during the day and 26 °C at night, with relative humidity at 50% for both day and night. Fifty days after sowing, each pot was scanned with an X-ray computed tomography (CT) scanner (InspeXio SMX-225CT FPD HR Plus; Shimadzu Co., Kyoto, Japan), at a tube voltage of 255 kV and current of 500 μA. RSA within the pot was non-destructively visualized and quantified using RSApaddy3D software (Teramoto and Uga, 2025).

### Statistical analyses

Statistical analyses were performed using the general linear model in SPSS (version 23.0; IBM Corp., Armonk, NY, USA). Two-way ANOVA was conducted to evaluate the effects of environment, genotype, and their interactions on the measured variables across eight environments (four sites×two fertilizer treatments) in 2023 and six environments (three sites×two fertilizer treatments) in 2024. Environment, genotype, and their interactions were considered fixed effects, whereas replication was considered a random effect. A post-hoc mean comparison was performed for the influence of environment and genotype using Tukey’s honest significant difference (HSD) test at *P*<0.05. To further investigate genotype-by-environment (G×E) interactions for grain yield in 2023, Finlay–Wilkinson regression analysis (Finlay and Wilkinson, 1963) was conducted. In this analysis, the grain yield of each genotype was regressed against the average yield of the 13 genotypes across each of the eight environments, used as an environmental index. Additionally, the eight environments were classified using Ward’s hierarchical clustering method (Ward, 1963), based on a transformed G×E interaction matrix for grain yield. First, a G×E matrix of mean grain yield (*Y*_(g,e)_) was constructed, where rows represented environments and columns represented genotypes. To isolate the interaction effect (*Y*_(G×E)_), the additive main effects of environments (*Y*_(e)_) and genotypes (*Y*_(G)_) were removed, and the grand mean (*Y*_mean_) was added using double centering as follows:


$$Y_{\left(G\times E\right)}=\;Y_{\left(G,\;E\right)}-Y_{\left(E\right)}-\;Y_{\left(G\right)}+\;Y_{\mathrm{mean}}$$

This transformation set both row and column means to zero, leaving only the G×E interaction effect (Wade *et al.*,1999). Euclidean distances among environments were then calculated based on the transformed matrix. Subsequently, environments were grouped using Ward’s hierarchical clustering method, which minimizes within-cluster variance in Euclidean space. The resulting clusters were identical to those obtained using *k*-means clustering (*k*=3), indicating consistent grouping results. Within each resulting cluster, Tukey’s HSD test was used to detect genotypic differences.

## Results

### Grain yield of IR64 and 12 NILs/PYLs in 2023

In 2023, grain yield was significantly influenced by the environment, genotype, and their interaction (Fig. 4A). Across the eight environments, the mean grain yield of the 13 genotypes widely varied, ranging from 1.6 t ha^−1^ at Site 1 without fertilizer to 5.1 t ha^−1^ at Site 4 with fertilizer (Fig. 4B). Notably, when we focused on the four environments (four sites) without fertilizer application, the mean Olsen P (available P) in the 0–30 cm soil layers—where the majority of roots were distributed in a previous study (Oo *et al*., 2025)—showed a significant correlation with the mean grain yield (Supplementary Fig. S1). This result suggests that differences in the mean grain yield among the four sites may be primarily attributable to differences in available P levels and that fertilizer application increased grain yield at each site. Next, the significant effects of genotype and its interaction with the environment prompted the use of Finlay–Wilkinson regression analysis to further assess grain yield variation. The analysis revealed that regression slopes differed among genotypes, and genotypic differences in grain yield increased with the environmental index (average yield among the 13 genotypes per environment), confirming a strong interaction between environment and genotype (Fig. 4C). Based on the interaction results, cluster analysis categorized the eight environments into three clusters (Clusters I, II, and III) (Fig. 4B, C). Cluster I was characterized by low yield levels (1.6–2.1 t ha^−1^), Cluster II by moderate yields (2.7–3.9 t ha^−1^), and Cluster III by high yields of >4 t ha^−1^ (4.1–5.1 t ha^−1^) (Supplementary Table S2). In Cluster I, no significant differences in mean grain yield were observed between IR64 and NILs/PYLs, although *DRO1*+*qsor1*-PYL tended to yield higher than IR64 (Fig. 4D). In Cluster II, *DRO1*+*qsor1*-PYL produced a significantly higher grain yield than IR64, with an increase of 23%. No other NILs/PLYs showed a significant difference compared with IR64. In Cluster III, the mean grain yield of *DRO1*+*qsor1*-PYL was higher than IR64 by 16%. In contrast, *qsor1*-NIL exhibited a 25% lower grain yield than IR64 in this cluster. Based on these results, four genotypes—IR64, *DRO1*-NIL, *qsor1*-NIL, and *DRO1*+*qsor1*-PYL—were selected for subsequent experiments in 2024.

**Fig. 4.**
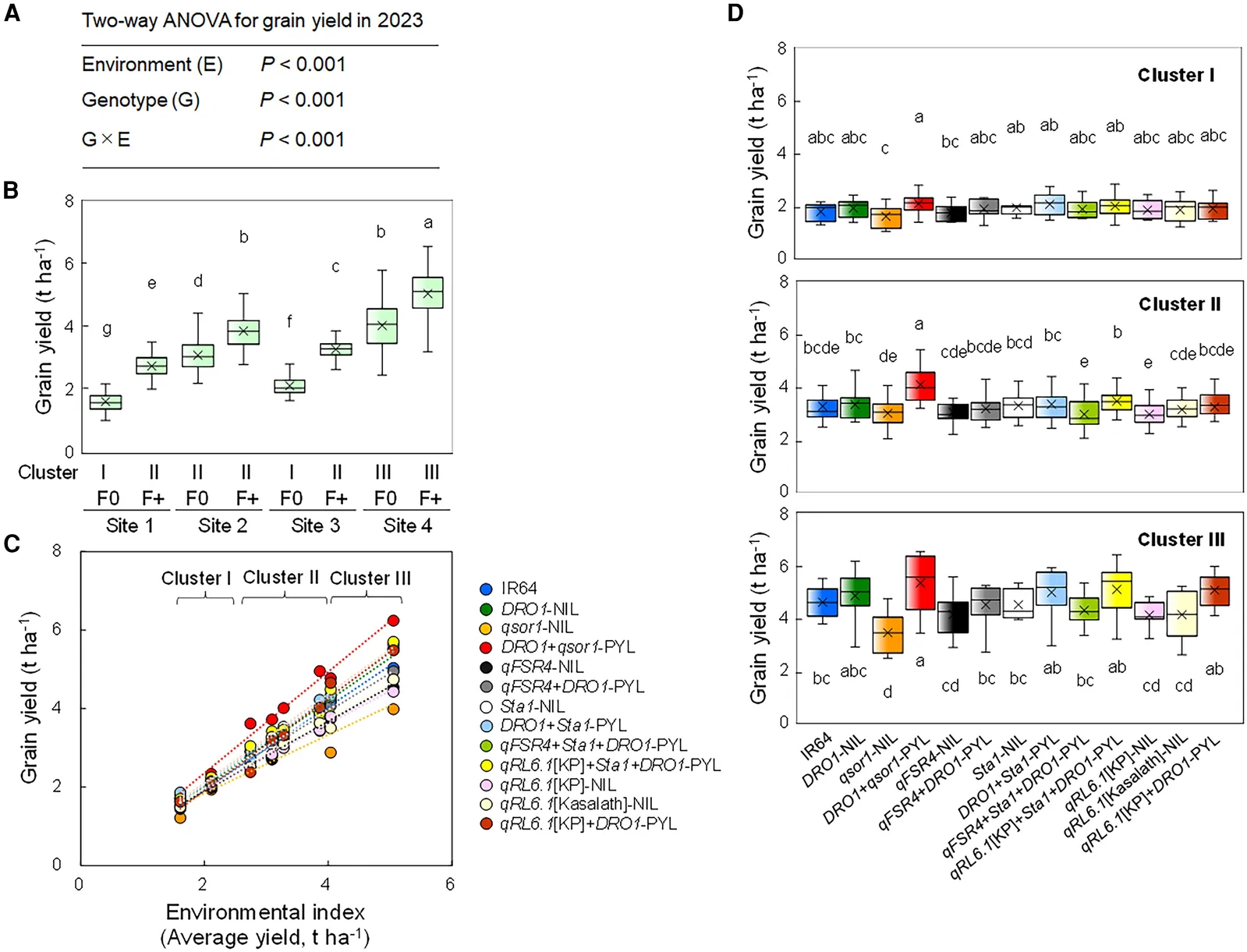
Rice grain yield comparisons in 2023 at the four experimental sites located in the central highlands of Madagascar. (A) Results of two-way ANOVA for grain yield among 13 rice genotypes across eight environments. (B) Comparison of mean grain yield across eight environments. F+ and F0 indicate fertilizer-applied and non-fertilizer conditions, respectively. The eight environments were grouped into three clusters (Clusters I, II, and III) based on hierarchical Ward’s method using interaction variables of grain yield. In the box plots, the cross indicates the mean, the central horizontal line shows the median, the box represents the interquartile range, and the whiskers indicate the maximum and minimum values. Different lowercase letters denote statistically significant differences among environments at the 5% significance level, as determined by Tukey’s honest significant difference test. (C) Finlay–Wilkinson regression analysis of grain yield. Grain yield of each genotype was plotted against the average yield of all 13 genotypes in each environment (environmental index). Dotted lines represent regression lines for individual genotype. Three clusters generated using Ward’s method are shown as Clusters I, II, and III. (D) Comparison of mean grain yield among 13 rice genotypes within each cluster. Different lowercase letters indicate significant differences among genotypes at 5% level based on Tukey’s honest significant difference test.

### Phenology, grain yield, and yield components of IR64, *DRO1*-NIL, *qsor1*-NIL, and *DRO1*+*qsor1*-PYL in 2024

In 2024, the mean grain yield among the four genotypes ranged from 2.4 t ha^−1^ at Site 3 without fertilizer to 6.2 t ha^−1^ at Site 4 with fertilizer across six environments (Fig. 5A). Site 3 without fertilizer was categorized into Cluster I in 2023, where *DRO1*+q*sor1*-PYL did not outyield IR64, while the other five environments were categorized into Clusters II and III in 2023, where *DRO1*+q*sor1*-PLY had consistently outperformed IR64. Therefore, we separately analysed the genotypic differences in relevant traits potentially contributing to this yield increase between Site 3 without fertilizer and the other five environments.

**Fig. 5.**
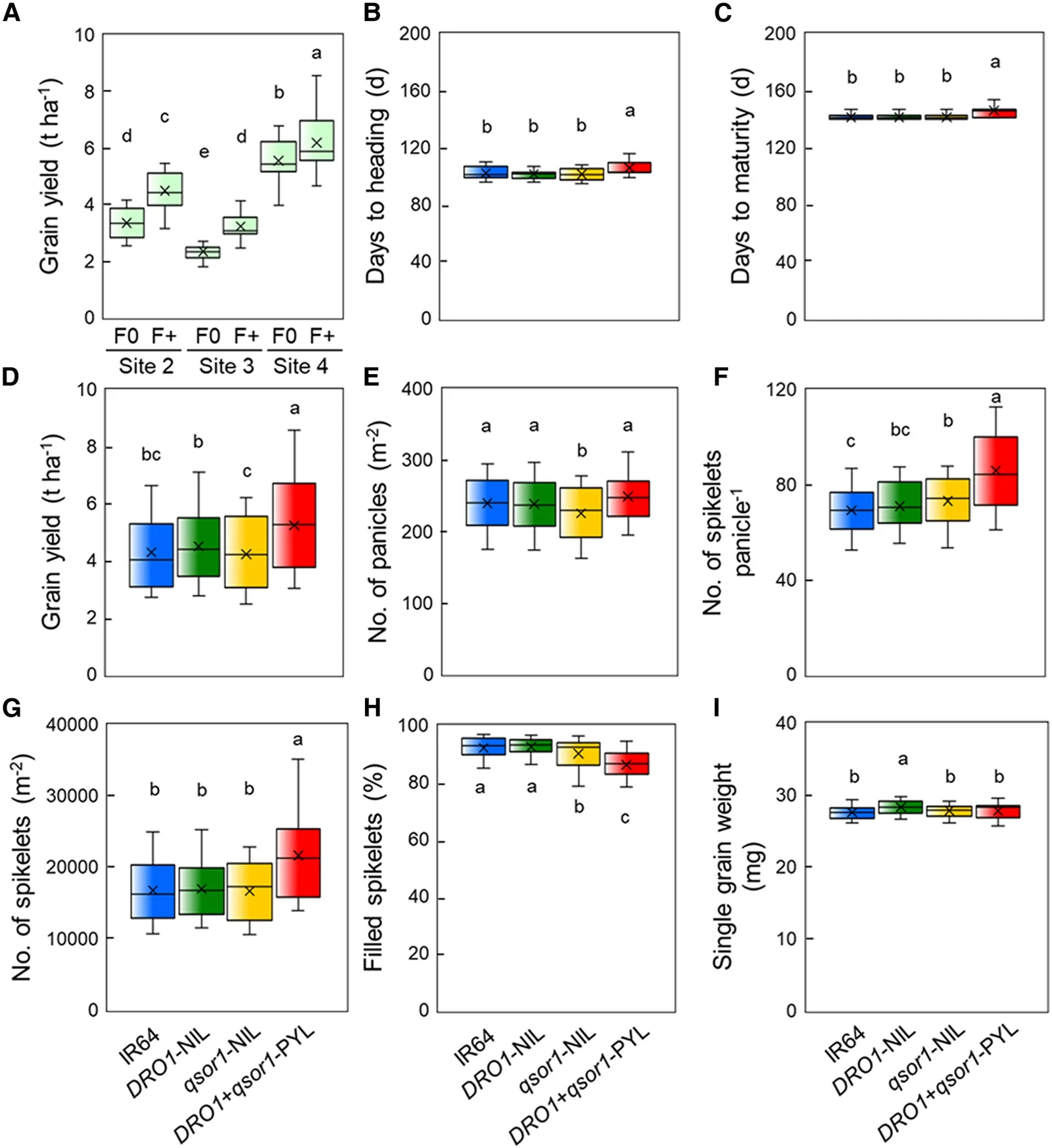
Rice grain yield comparisons in 2024 at the three experimental sites located in the central highlands of Madagascar. (A) Mean grain yield across six environments. F+ and F0 indicate fertilizer-applied and non-fertilizer conditions, respectively. Different lowercase letters denote statistically significant differences among environments at the 5% significance level, as determined by Tukey’s honest significant difference test. (B–I) Comparisons of phenology (B, C), grain yield (D), and yield components (E–I) among four genotypes grown across the five environments except for Site 3 without fertilizer. In each box plot, the cross represents the mean, the central horizontal bar indicates the median, the box shows the interquartile range, and the whiskers denote the maximum and minimum values. Different lowercase letters denote statistically significant differences among genotypes at the 5% significance level, as determined by Tukey’s honest significant difference test.

In the five environments, days to heading was similar among IR64, *DRO1*-NIL, and *qsor1*-NIL, whereas that of *DRO1*+*qsor1*-PYL was 3.5 d later than that of IR64 (Fig. 5B). Similarly, days to maturity was comparable among IR64, *DRO1*-NIL, and *qsor1*-NIL, whereas *DRO1*+*qsor1*-PYL matured 4.6 d later than IR64 (Fig. 5C). Consistent with results from 2023, *DRO1*+*qsor1*-PYL achieved the highest overall mean grain yield in 2024 (5.2 t ha^−1^), followed by *DRO1*-NIL (4.5 t ha^−1^), IR64 (4.3 t ha^−1^), and *qsor1*-NIL (4.2 t ha^−1^) (Fig. 5D). The grain yield of *DRO1*+*qsor1*-PYL was 22% higher than that of IR64. Although the mean number of panicles per unit area was comparable between *DRO1*+*qsor1*-PYL and IR64 (Fig. 5E), the mean number of spikelets per panicle was significantly greater for *DRO1*+*qsor1*-PYL (86) than for IR64 (69) representing a 24% increase (Fig. 5F). As a result, total spikelet production of *DRO1*+*qsor1*-PYL (21 691 spikelets m^−2^) was 29% higher than that of IR64 (16 781 spikelets m^−2^) (Fig. 5G). In contrast, the percentage of filled spikelets was lowest for *DRO1*+*qsor1*-PYL (86.3%), followed by *qsor1*-NIL (90.3%), both significantly lower than IR64 (92.2%; Fig. 5H). The single grain weight was not significantly different between IR64 and *DRO1*+*qsor1*-PYL (Fig. 5I).

At Site 3 without fertilizer, days to heading and maturity were similar among IR64, *DRO1*-NIL, and *qsor1*-NIL, whereas *DRO1*+*qsor1*-PYL headed 5 d later and matured 7 d later than IR64 (Supplementary Fig. S2A, B). In contrast to the five environments, no significant difference in grain yield was observed among the four genotypes (Supplementary Fig. S2C). The number of panicles per unit area and the number of spikelets per panicle were comparable among the four genotypes (Supplementary Fig. S2D, E). As a result, unlike in the five environments, no significant difference was observed between IR64 and *DRO1*+*qsor1*-PYL (Supplementary Fig. S2F). The percentage of filled spikelets was lowest in *DRO1*+*qsor1*-PYL, followed by *qsor1*-NIL, and both were significantly lower than that in IR64 (Supplementary Fig. S2G). The single grain weight was not significantly different among the four genotypes (Supplementary Fig. S2H).

### Root phenotype of IR64, *DRO1*-NIL, *qsor1*-NIL, and *DRO1*+*qsor1*-PYL

Total root biomass—from the soil surface to a depth of 30 cm—did not differ among the four genotypes in the five environments and at Site 3 without fertilizer (Fig. 6A; Supplementary Fig. S4A). However, the basket method revealed distinct effects of the *DRO1* and *qsor1* alleles under nutrient-poor soils of the central highlands of Madagascar (Supplementary Fig. S3). Specifically, *DRO1*-NIL exhibited a higher proportion of roots at the bottom of the basket than IR64, whereas *qsor1*-NIL showed a greater proportion of roots at the basket surface in the five environments and at Site 3 without fertilizer (Fig. 6B; Supplementary Fig. S4B). Notably, *DRO1*+*qsor1*-PYL displayed a dimorphic root phenotype (Ogawa *et al*., 2014), with increased root proportions at both the bottom and surface of the basket, confirming the pyramiding effects of *DRO1* and *qsor1*. Consistent with these findings, three-dimensional imaging with X-ray CT in pot experiments visually confirmed similar genotypic differences in root distribution patterns at 50 d after sowing (Supplementary Fig. S5). Regardless of NPK fertilizer application, *DRO1*-NIL developed more roots in soil layers deeper than 10 cm than IR64, whereas *qsor1*-NIL developed more roots near the soil surface (Fig. 6C; Supplementary videos S1–S8). No roots were observed below 10 cm in *qsor1*-NIL. In contrast, *DRO1*+*qsor1*-PYL developed greater root mass in both deep and surface layers than IR64.

**Fig. 6.**
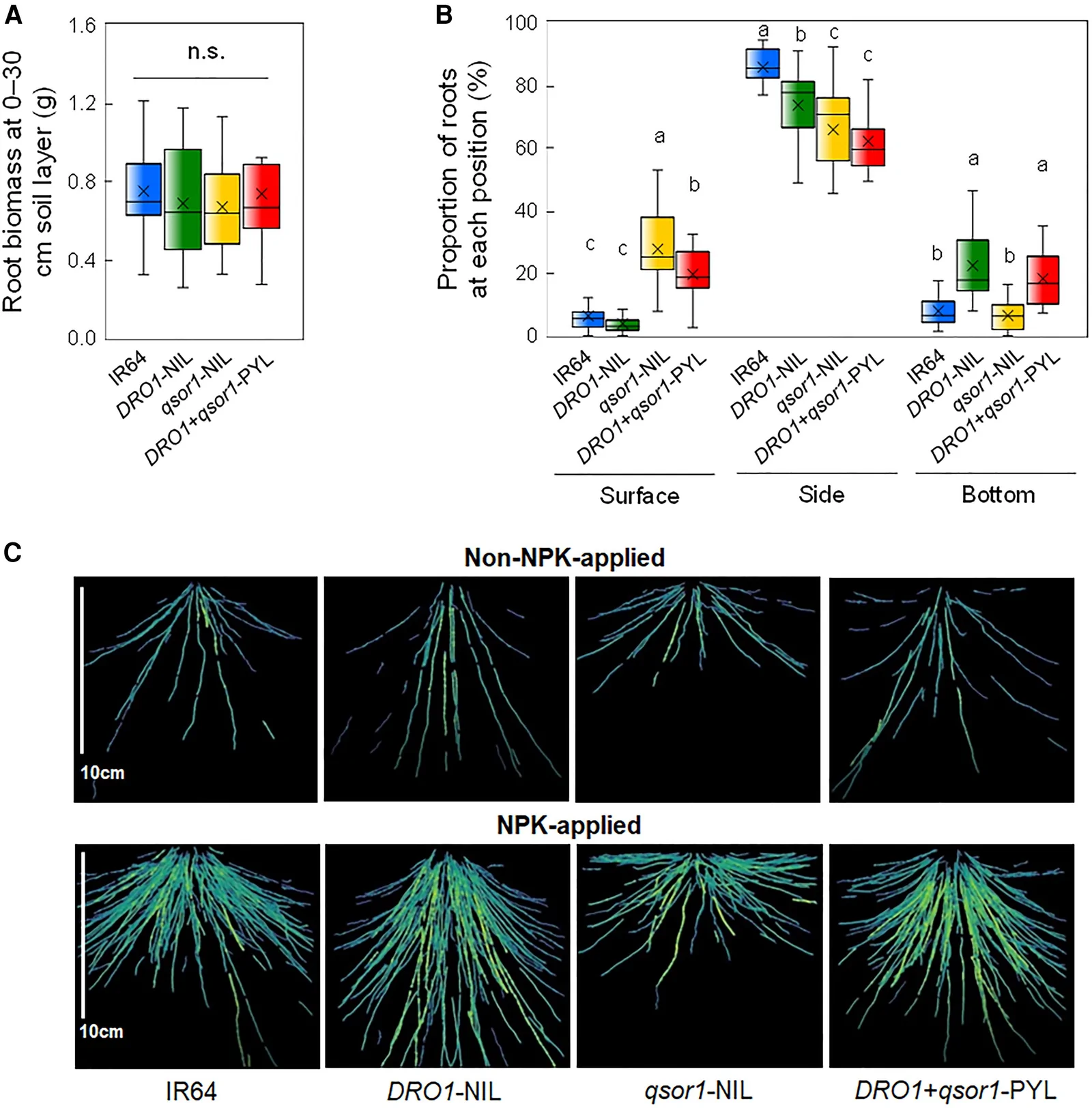
Comparisons of root phenotypes among four genotypes in 2024 across the five environments, except for Site 3 without fertilizer, in the central highlands of Madagascar. (A) Root weight collected from the 0–30 cm soil layer using a monolith sampler. The cross in each box represents the mean, the central horizontal bar shows the median, the box represents the interquartile range, and the whiskers show the maximum and minimum values. n.s. denotes no significant differences among genotypes at the 5% significance level, as determined by Tukey’s honest significant difference test. (B) Proportions of roots located on the surface, side, and bottom of baskets placed in paddy fields in Madagascar. Different lowercase letters denote statistically significant differences among genotypes at the 5% significance level, as determined by Tukey’s honest significant difference test. (C) Three-dimensional images of root system architecture in pot-grown plants at 50 d after sowing under NPK-applied or non-applied conditions.

### Nutrient uptake and aboveground biomass production

In the five environments, *DRO1*+*qsor1*-PYL exhibited the highest overall N uptake at maturity among the four genotypes, significantly exceeding that for IR64 by 10% (Fig. 7A). Similarly, P uptake was significantly higher for *DRO1*+*qsor1*-PYL than for the other genotypes, with a 16% increase compared to IR64 (Fig. 7B). Aboveground biomass production followed a similar trend: *DRO1*+*qsor1*-PYL had the highest biomass, whereas *qsor1*-NIL had the lowest (Fig. 7C). *DRO1*+*qsor1*-PYL produced significantly higher (by 18%) aboveground biomass than IR64, indicating that enhanced nutrient acquisition and biomass accumulation is associated with the pyramided root traits.

**Fig. 7.**
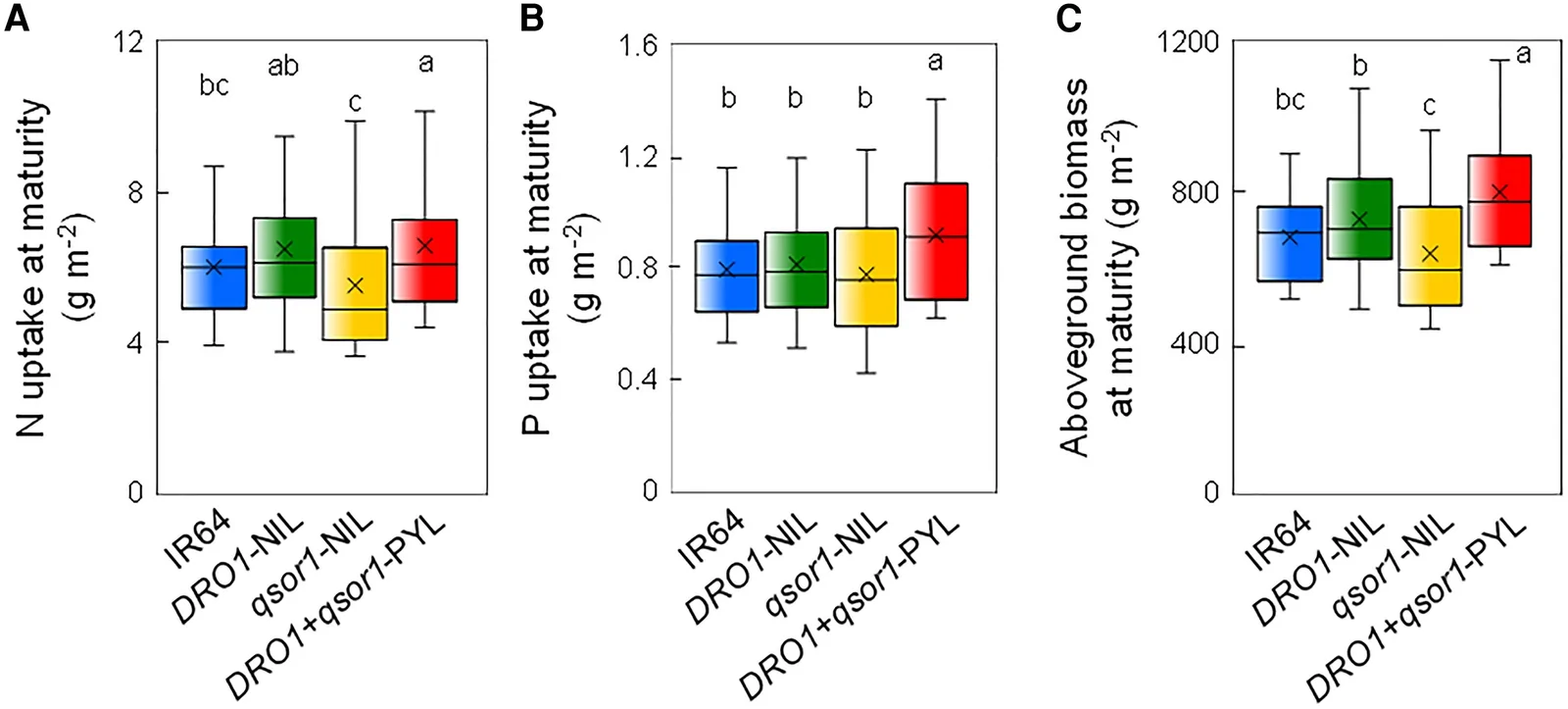
Comparisons of N and P uptake (A, B) and biomass (C) at maturity among four genotypes in 2024 across the five environments, except for Site 3 without fertilizer, in the central highlands of Madagascar. The cross in each box represents the mean, the central horizontal bar shows the median, the box represents the interquartile range, and the whiskers show the maximum and minimum values. Different lowercase letters indicate statistically significant differences among genotypes at the 5% significance level, as determined by Tukey’s honest significant difference test.

In contrast, neither N uptake, P uptake, nor aboveground biomass significantly differed among the four genotypes in Site 3 without fertilizer (Supplementary Fig. S6A–C).

## Discussion

In this study, total N and available P contents in the paddy soils of the central highlands of Madagascar did not exhibit consistent depth gradients as observed in upland soils; however, mean grain yield increased with higher available P levels and fertilizer application. Under the soil conditions, we demonstrated that the dimorphic-root phenotype—characterized by a combination of deeper and shallower roots and conferred by the *DRO1*+*qsor1*-PYL allele—improved rice grain yield by 16–23% at yield levels of 2.7 to 5.1 t ha^−1^ defined as Clusters II and III. The potential benefits of the dimorphic-root system have been proposed to be efficient uptake of N and water under upland conditions in maize (Dathe *et al*., 2016); however, to our knowledge, no studies have demonstrated its importance in nutrient-deficient paddy fields. However, the yield advantage of the dimorphic-root system was less pronounced at sites with yields below 2.1 t ha^−1^ defined as Cluster I, where available P in soils was extremely low and no vertical movement of N (no abundant N in the subsoil) occurred due to minimal fertilizer input. These findings clearly indicate that expanding root distribution into both the topsoil and subsoil constitutes an effective RSA ideotype for enhancing rice productivity in nutrient-deficient paddy fields, regardless of nutrient gradients with depth. However, this does not hold true for paddy fields with severe nutrient limitations, where an expanded root distribution does not necessarily enhance nutrient uptake.

The dimorphic expansion of root distribution in *DRO1*+*qsor1*-PYL, observed via the basket method and X-ray CT scans, enhanced both N and P uptake at the yield levels of Clusters II and III. This increased nutrient uptake likely contributed to higher grain yields, primarily via increased aboveground biomass and spikelet density (spikelets m^−2^). In a previous study, grain yield of *DRO1*-NIL increased under nutrient-rich paddy field conditions (Arai-Sanoh *et al*., 2014). In the present study, *DRO1*-NIL also tended to outperform IR64 in yield at Clusters II and III, though the difference was not statistically significant. These findings suggest that deeper rooting conferred by *DRO1* alone is insufficient to improve yields under N- and P-deficient paddy fields. Conversely, *qsor1*-NIL exhibited the lowest yield and N uptake, indicating a possible limitation in accessing subsoil nutrients. Although shallow roots are considered advantageous when P typically accumulates in the topsoil (Oo *et al*., 2021; Uga, 2021), they may be less effective in capturing nutrients from deeper layers. X-ray CT scans revealed that most roots of *qsor1*-NIL were confined to the upper 10 cm of soil. Notably, available P was not always lower in the subsoil than in the topsoil (Fig. 3), possibly due to repeated puddling and long-term absence of fertilization, which may have altered the soil nutrient gradient. These findings suggest that deeper roots may be essential for efficient uptake of both P and N from subsoil layers, particularly under long-term unfertilized conditions. Moreover, differences in grain yield among the tested genotypes were generally consistent across fertilizer treatments, with or without surface-applied NPK, highlighting the relevance of root system traits. These findings suggest that dimorphic root distribution—expanding into both topsoil and subsoil—is critical for efficient N and P acquisition, regardless of NPK application, in nutrient-deficient paddy fields. Collectively, these results highlight the agronomic potential of combining deeper and shallower root traits, and therefore the development of rice cultivars with this dimorphic RSA is essential for future breeding programs targeting nutrient-deficient soils in SSA.

The yield improvement observed for the *DRO1*+*qsor1*-PYL at the yield levels of Clusters II and III was attributed to an increase in spikelet number per panicle, rather than an increase in panicle number per square meter. Rice plants initiate tillering early in the vegetative stage, a process promoted by nutrient uptake (Nakano and Morita, 2009; Takai *et al*., 2024a). The similar panicle number between IR64 and *DRO1*+*qsor1*-PYL indicates no increase in N and P uptake in *DRO1*+*qsor1*-PYL during the early vegetative stage, although the X-ray CT scans showed that RSA associated with *DRO1* and *qsor1* had already begun to develop at that time. Enhanced N and P uptake may have occurred during the late vegetative or reproductive stage, after the RSA was fully established and tillering had ceased. Consequently, *DRO1*+*qsor*1-PYL likely increased spikelet number per panicle rather than panicle number. This is consistent with the observation at the yield level of Cluster I, where N and P uptake in *DRO1*+*qsor*1-PYL did not increase compared with that of IR64, resulting in no difference in spikelet number per panicle and, consequently, grain yield.

In this study, *DRO1*+*qsor1*-PYL exhibited heading and maturity 3.5 and 4.6 d later than IR64, respectively, at the yield levels of Cluster II and III. Delayed heading and maturity can extend the total growth period and potentially increase biomass and grain yield (Xue *et al*., 2008). However, such delay in heading and maturity extends the overall growth period by only approximately 3%, which is insufficient to account for the 16–23% yield increase observed in *DRO1*+*qsor1*-PYL. Moreover, further delays of 5 and 7 d in heading and maturity were observed in *DRO1*+*qsor1*-PYL at the yield level of Cluster I, which did not result in any increase in grain yield. Therefore, the effect of delay in heading and maturity on grain yield appears to be minimal, if present at all. Nevertheless, further studies are needed to assess the influence of late heading and maturity on yield improvement in *DRO1*+*qsor1*-PYL by eliminating QTLs linked to heading delay from the genome.

Additionally, *qFSR4*, *Sta1*, and *qRL6.1* did not contribute to an increase in grain yield in this study. *qFSR4* enhances root volume, *qRL6.1* promotes root elongation, and *Sta1* increases root thickness (Obara *et al*., 2010; Uga *et al*., 2010; Ding *et al*., 2011), indicating that these QTLs may lead to an increase in the number of cortical cells in roots. An increase in cortical cell number elevates the respiratory burden of the root cortex and thereby raises energy costs, potentially leading to a trade-off in nutrient acquisition efficiency (Lynch, 2013). The formation of root cortical aerenchyma—through the conversion of living cortical tissue to air spaces—has been proposed as a strategy to decrease respiration and consequently reduce energy expenditure in roots (Fan *et al*., 2003). In contrast, the altered root growth angle caused by *DRO1* and *qsor1* appears to have required little additional energy, as it was independent of the change in root biomass and thus involves minimal alteration in the number of cortical cells. Therefore, *DRO1* and *qsor1* may confer an advantage in energy efficiency in nutrient-deficient paddy fields.

In conclusion, the combination of deeper and shallower roots conferred by *DRO1* and *qsor1* expanded root distribution into both the topsoil and subsoil, enhancing N and P uptake, increasing aboveground biomass and spikelet number per panicle, and ultimately improving rice yield by 16–23% in the nutrient-deficient paddy fields, except under severe nutrient-limiting conditions. This expanded root distribution represents a promising RSA ideotype for nutrient-deficient paddy fields in SSA and could serve as a valuable breeding target to enhance rice production in this region and other agroecosystems facing similar constraints. The increase in spikelet number per panicle associated with *DRO1* and *qsor1* also indicates the potential for further yield gains via an increase in panicle number, which may be achieved by pyramiding with *MP3*, a QTL known to promote tillering under nutrient-deficient conditions (Takai *et al*., 2021). Pyramiding *DRO1*, *qsor1*, and *MP3* could enhance both above- and belowground plant architecture, and eventually contribute to improving gain yield even under severe nutrient-limiting conditions. We are currently focusing on introducing *MP3*, *qsor1*, and *DRO2*, another deep-rooting QTL (Uga *et al*., 2013b), into the genetic background of X265—a leading rice cultivar in Madagascar. Pyramiding the QTLs could provide new insights into the combined effects of above- and belowground trait improvements and ultimately accelerate rice yield gains under nutrient-deficient conditions across SSA, including Madagascar.

## Supplementary Material

erag043_Supplementary_Data

## Data Availability

All data supporting the findings of this study are available in the paper and its supplementary data published online.

## Supplementary data

The following supplementary data are available at *JXB* online.

Fig. S1. Correlation coefficients between soil chemical properties (averaged across 0–30 cm depth) and the mean grain yield of 13 genotypes across four sites without fertilizer application (F0).

Fig.S2. Comparison of rice grain yield among four genotypes grown at Site 3 without fertilizer application in 2024.

Fig. S3. Images of root distribution among four genotypes taken from above and below the baskets.

Fig. S4. Comparison of root phenotypes among four genotypes at Site 3 without fertilizer application in 2024.

Fig. S5. Image of pot plants grown in the nutrient-deficient soil with and without NPK application just before X-ray CT scan.

Fig. S6. Comparison of N and P uptake and biomass at maturity among four genotypes at Site 3 without fertilizer application in 2024.

Table S1. Monthly averages of daily mean temperature and daily solar radiation during rice growing periods.

Table S2. Grain yield of 13 genotypes under two fertilizer treatments at the four experimental sites in 2023.

Video S1. Animation of a three-dimensional rendering of the root system architecture in IR64 grown without fertilizer application.

Video S2. Animation of a three-dimensional rendering of the root system architecture in *DRO1*-NIL grown without fertilizer application.

Video S3. Animation of a three-dimensional rendering of the root system architecture in *qsor1*-NIL grown without fertilizer application.

Video S4. Animation of a three-dimensional rendering of the root system architecture in *DRO1*+*qsor1*-PYL grown without fertilizer application.

Video S5. Animation of a three-dimensional rendering of the root system architecture in IR64 grown with fertilizer application.

Video S6. Animation of a three-dimensional rendering of the root system architecture in *DRO1*-NIL grown with fertilizer application.

Video S7. Animation of a three-dimensional rendering of the root system architecture in *qsor1*-NIL grown with fertilizer application.

Video S8. Animation of a three-dimensional rendering of the root system architecture in *DRO1*+*qsor1*-PYL grown with fertilizer application.

## Abbreviations

CT: computed tomography
NIL: near-isogenic line
PYL: pyramiding line
QTL: quantitative trait locus
RSA: root system architecture
SSA: sub-Saharan Africa
