# Supplementary material for: Dimorphic-root ideotype demonstrates increased rice yield in nutrient-deficient paddy fields in Madagascar
Source: J Exp Bot. 2026 Jan 27;77(8):2565–77. doi: 10.1093/jxb/erag043 (PMC13728848; doi:10.1093/jxb/erag043)
Supplement: Supplementary file 1 [file erag043_supplementary_data.zip › jexbot317125-file001.pdf]

Supplementary Table S1. Monthly averages of daily mean temperature and daily solar radiation during rice growing periods

	Site 1 and 2		Site 3 and 4	
	2023	2024	2023	2024
Mean temperature (˚C)				
Nov	20.6	21.7	21.6	22.1
Dec	20.9	22.7	22.0	22.7
Jan	20.1	21.7	21.1	21.7
Feb	21.2	21.4	22.2	21.6
Mar	20.7	21.5	21.7	21.9
Apr	20.1	21.4	21.5	22.2
May	18.3	19.0	19.9	19.6
Solar radiation (MJ m^{-2} day^{-1})				
Nov	27.0	18.3	25.3	21.3
Dec	26.8	18.0	24.9	21.0
Jan	20.9	16.6	19.1	21.4
Feb	26.7	16.4	25.1	16.1
Mar	23.6	19.0	21.3	21.1
Apr	23.5	24.5	23.0	21.3
May	22.0	21.5	20.1	19.5

Supplementary Table S2. Grain yield of 13 genotypes under two fertilizer treatments at the four experimental sites in 2023

Genotype	Site	1		2		3		4	
	Fertilizer	F0	F+	F0	F+	F0	F+	F0	F+
	Cluster	I	II	II	II	I	II	III	III
IR64	Yield (t ha^{-1})	1.5	2.7	3.1	4.1	2.0	3.1	4.2	5.0
DRO1-NIL		1.6	2.9	3.1	3.8	2.1	3.4	4.2	5.5
qsor1-NIL		1.2	2.5	2.8	3.4	1.9	3.3	2.9	4.0
DRO1+*qsor1*-PYL		1.8	3.6	3.7	5.0	2.3	4.0	4.8	6.2
qFSR4-NIL		1.4	2.7	2.7	3.5	2.0	3.2	3.7	4.5
qFSR4+*DRO1*-PYL		1.7	2.9	2.8	3.9	2.0	3.1	4.1	4.9
Sta1-NIL		1.7	2.7	3.1	3.8	2.1	3.5	4.3	4.7
DRO1+*Sta1*-PYL		1.9	2.7	3.3	4.2	2.2	3.1	4.3	5.6
qFSR4+*Sta1*+*DRO1*-PYL		1.6	2.4	2.9	3.6	2.1	3.0	3.9	4.7
qRL6.1[KP]+*Sta1*+*DRO1*-PYL		1.7	3.0	3.4	3.9	2.3	3.5	4.5	5.7
qRL6.1[KP]-NIL		1.6	2.5	2.8	3.5	2.1	3.0	3.8	4.4
qRL6.1[Kasalath]-NIL		1.5	2.5	3.3	3.6	2.2	3.1	3.5	4.7
qRL6.1[KP]+*DRO1*-PYL		1.6	2.4	3.2	4.0	2.1	3.3	4.7	5.5
Average		1.6	2.7	3.1	3.9	2.1	3.3	4.1	5.1

F+ and F0 indicate fertilizer-applied and non-fertilizer conditions, respectively.

Eight environments (four sites × two fertilizer treatments) were classified into the three cluster groups, based on a G × E interaction matrix for grain yield.

Correlation coefficients

	Organic C	Total N	Olsen P	pH
Yield	-0.439	-0.301	0.988*	0.431

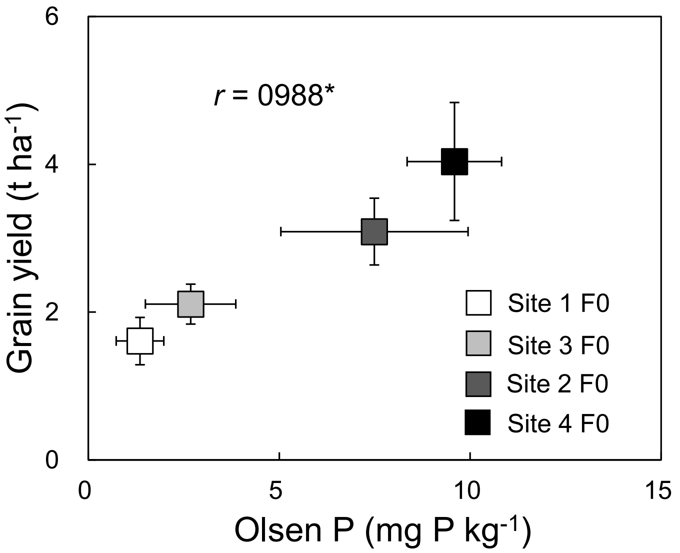

Supplementary Fig. S1. Correlation coefficients between soil chemical properties (averaged across 0–30 cm depth) and the mean grain yield of 13 genotypes across four sites without fertilizer application (F0). * indicates significant correlations at the 5% significance level.

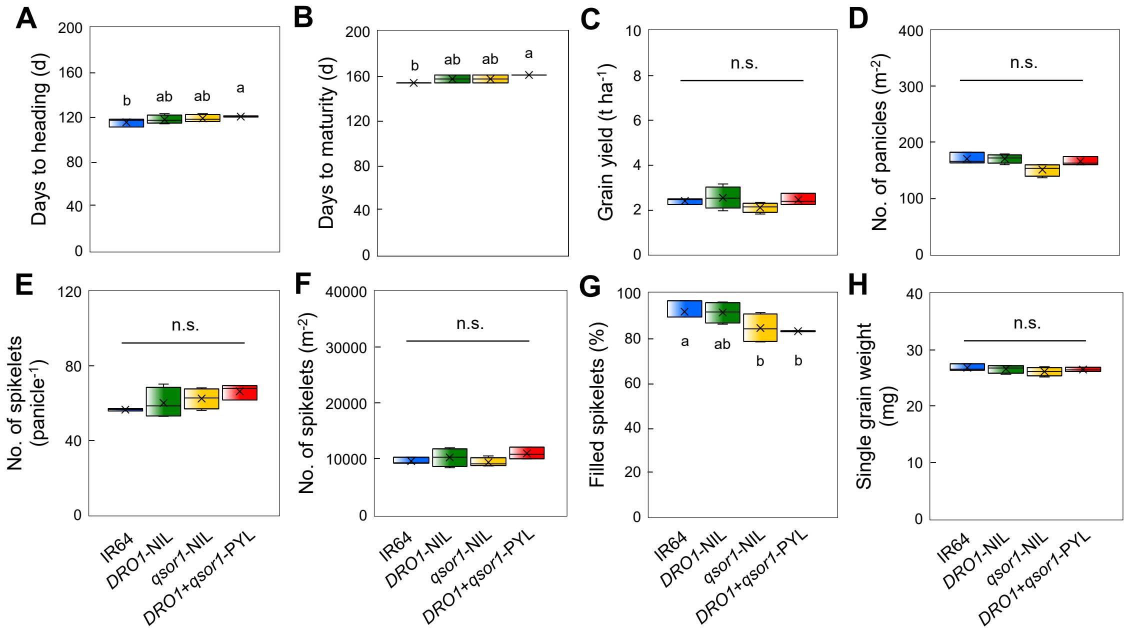

Supplementary Fig. S2. Comparison of rice grain yield among four genotypes grown at Site 3 without fertilizer application in 2024. Comparisons of phenology (A, B), grain yield (C), and yield components (D–H). In each box plot, the cross represents the mean, the central horizontal bar indicates the median, the box shows the interquartile range, and the whiskers denote the maximum and minimum values. n.s. denotes no significant differences among genotypes at the 5% significance level, as determined by Tukey's honest significant difference test. Different lowercase letters denote statistically significant differences among genotypes.

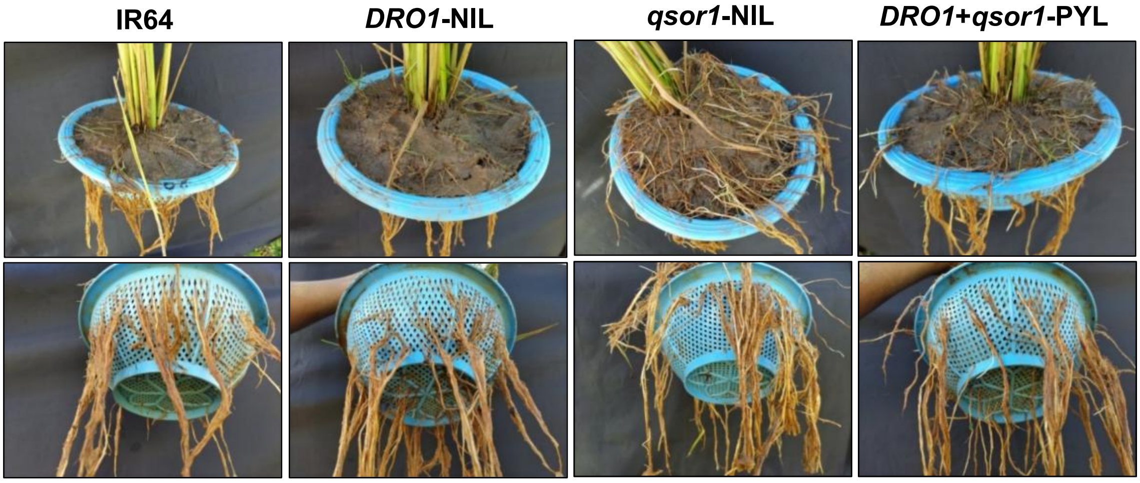

Supplementary Fig. S3. Images of root distribution among four genotypes taken from above and below the baskets.

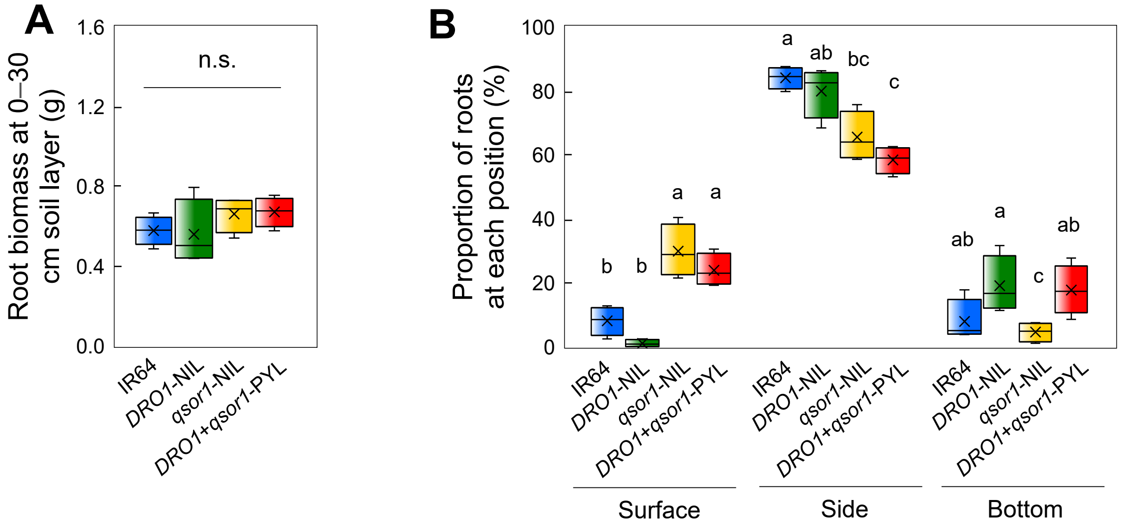

Supplementary Fig. S4. Comparisons of root phenotypes among four genotypes in 2024 at Site 3 without fertilizer application in 2024. (A) Root weight collected from the 0–30-cm soil layer using a monolith sampler. The cross in each box represents the mean, the central horizontal bar shows the median, the box represents the interquartile range, and the whiskers show the maximum and minimum values. n.s. denotes no significant differences among genotypes at the 5% significance level, as determined by Tukey's honest significant difference test. (B) Proportions of roots located on the surface, side, and bottom of baskets placed in paddy fields in Madagascar. Different lowercase letters denote statistically significant differences among genotypes at the 5% significance level, as determined by Tukey's honest significant difference test.

.

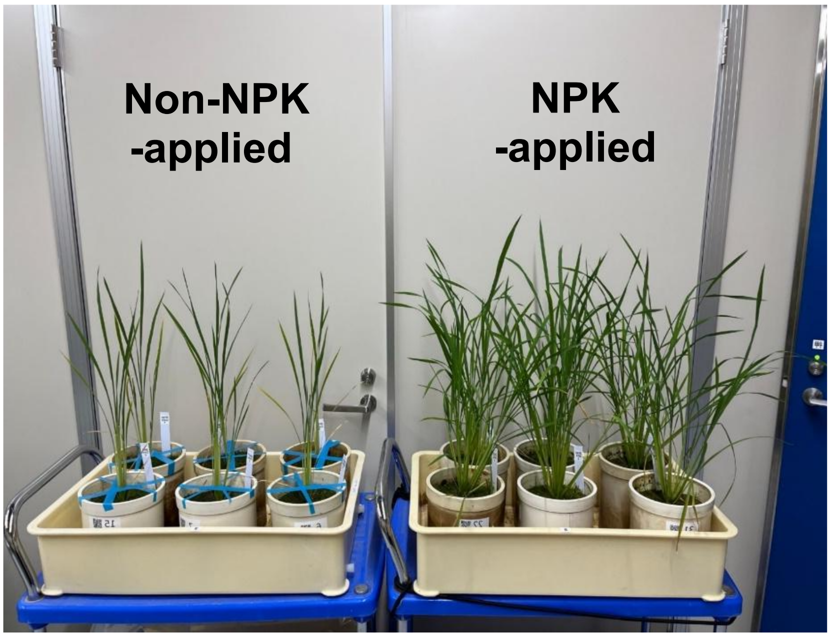

Supplementary Fig. S5. Image of pot plants grown in the nutrient-deficient soil with and without NPK application just before X-ray CT scan.

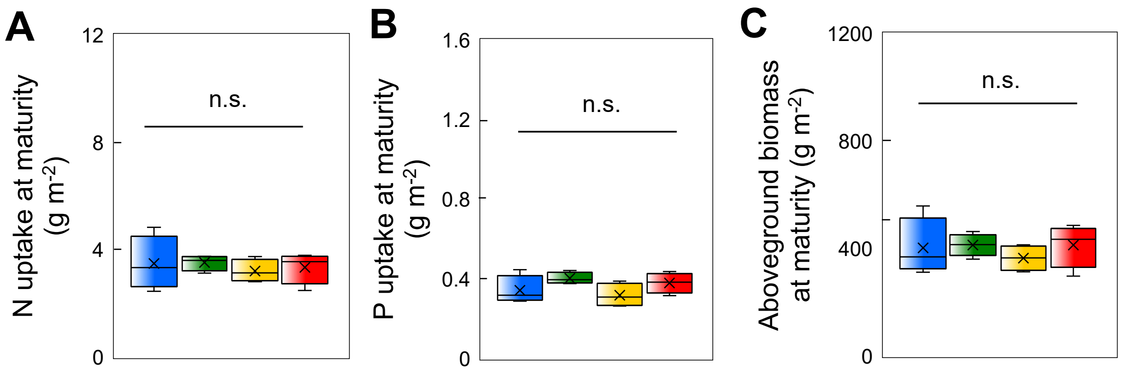

Supplementary Fig. S6. Comparisons of N and P uptake (A, B) and biomass (C) at maturity among four genotypes in 2024 at Site 3 without fertilizer application in 2024. The cross in each box represents the mean, the central horizontal bar shows the median, the box represents the interquartile range, and the whiskers show the maximum and minimum values. n.s. indicates no significant differences among genotypes at the 5% significance level, as determined by Tukey's honest significant difference test.
